# Impaired redox regulation of estrogen metabolizing proteins is important determinant of human breast cancers

**DOI:** 10.1186/s12935-019-0826-x

**Published:** 2019-05-15

**Authors:** Smarajit Maiti, Aarifa Nazmeen

**Affiliations:** 1 Dept. of Biochemistry, Cell & Molecular Therapeutics Lab, Oriental Institute of Science & Technology, Midnapore, 721101 India; 2 Department of Biochemistry and Biotechnology, Cell & Molecular Therapeutics Lab, OIST, Midnapore, 721102 India

**Keywords:** Estrogen, hSULT1E1, Estrogen sulfatase, Formylglycine generating enzyme, Gynecological cancers, ERα positive

## Abstract

Estrogen evidently involves critically in the pathogenesis of gynaecological-cancers. Reports reveal that interference in estrogen-signalling can influence cell-cycle associated regulatory-processes in female reproductive-organs. The major determinants that influence E2-signallings are estrogen-receptor (ER), estrogen-sulfotransferase (SULT1E1), sulfatase (STS), and a formylglycine-generating-enzyme (FGE) which regulates STS activity. The purpose of this mini review was to critically analyze the correlation between oxidative-threats and redox-regulation in the process of estrogen signalling. It is extensively investigated and reported that oxidative-stress is linked to cancer. But no definite mechanism has been explored till date. The adverse effects of oxidative-threat/free-radicals (like genotoxic-effects, gene-regulation, and mitochondrial impairment) have been linked to several diseases like diabetes/cardiovascular-syndrome/stroke and cancer. However, a significant correlation between oxidative-stress and gynaecological-cancers are repeatedly reported without pointing a definite mechanism. For the first time in our study we have investigated the relationship between oxidative stress and the regulation of estrogen via estrogen metabolizing proteins. Reports reveal that ER, SULT1E1, STS and FGE are target-molecules of oxidative-stress and may function differently in oxidizing and reducing environment. In addition, estrogen itself can induce oxidative-stress. This fact necessitates identifying the critical connecting events between oxidative-stress and regulation of estrogen-associated-molecules (ER, SULT1E1, STS, and FGE) that favors tumorigenesis/carcinogenesis. The current review focus is on unique redox-regulation of estrogen and its regulatory-molecules via oxidative-stress. This mechanistic-layout may identify new therapeutic-targets and open further scopes to treat gynecological-cancers more effectively.

## Introduction

The role of estrogen and several of its metabolites in the pathogenesis of breast cancer has been extensively discussed for the last few decades. An elevated level of estrogen and breast cancer has already been. The risk of developing breast cancer significantly increases due to early menarche, late menopause or estrogen replacement therapy (ERT) [1, 2]. Association of serum androgen with premenopausal breast cancer was supported by animal models also [3, 4]. International Agency for Research (IAR) and the National Toxicology Program of National Institute of Environmental Health Sciences (NIEHS) declared that endogenous/exogenous estrogens may be regarded as “human carcinogens” [5]. Steroids like estrogen and progestogens at an increased levels, increases cellular proliferation mostly in human breast tissue [6]. The linkage among estrogen level, rate of proliferation and the process of carcinogenesis strongly indicates the role of E2-ER dependent mechanism in breast cancers. There is a distinction between receptor-dependent and independent activity of estrogen which is best elucidated by experimental animal models using knock-out animal models. A study reports that 50% of mammary tumors arose within few months in the ERα+ animals versus longer periods in those without ERα (i.e. ERKO animals) [7, 8]. Therapeutic efficacies of tamoxifen or raloxifene in breast cancer demonstrate a role for ERα in breast carcinogenesis process [9]. Interestingly, 10 to 24% of BRCA1 carrying breast tumors were ERα+ in nature [10]. This percentage constitutes a vast number of affected populations worldwide.

### Estrogen synthesis and its regulations

Estradiol (E2) is the most potent form of estrogenic steroids than estriol (E3) and estrone (E1) that ovaries synthesize. Estradiol exerts the maximum range of estrogenic effects becoming a potent inducer of cancer [11]. Estrogen production in peripheral tissues is accomplished by the “sulfatase pathway’’ and the “aromatase pathway” [12]. Estrogen regulating biomolecules like estrogen sulfotransferase (SULT1E1), sulfatase (STS), estrogen receptor α (ERα) and estrogen receptor β (ERβ) directly or indirectly may affect the availability and activity of estrogen.

### Mode of action of estrogen (genomic pathway and non-genomic pathway)

The biological effects of estrogen are mediated through ligand activated transcription factors, estrogen receptors (ERα and ERß) [13]. There are three major modes of estrogen action, the direct genomic pathway where E2-ER binds to estrogen receptor like element (ERE) and causes expression of important genes. The genes expressed by direct genomic pathways are JUN, FOS, PGR, TP53, HRAS, Bcl2, BRCA1, CHAT, NQO1, CKB, LTF, SCGB1A1 etc. [14]. The indirect genomic pathway includes the binding of E2-ER to other transcription factor which binds to their specific elements and promotes gene expression. Estrogen receptors interaction with the activator protein1 (AP-1), signal transducer and activator of transcription (STATs), activator of transcription factor 2 (ATF-2)/c-Jun, Sp1, and nuclear factor kappa-light-chain-enhancer of activated B cells (NFκβ) are evident as the indirect genomic activity of estrogen. E2 and ERs via multiple response elements finely controls the transcriptional regulations of target genes. E2-ER via the non-classical pathways activates kinase pathways such as ERK, P38/MAPK, P13K, AKT, PLC, PKC, cyclic AMP/PKA that ultimately regulate transcription factors and their specific genes. GPER target, genes like c-fos is also involved in the progression of breast malignancies through the EGFR/MAPK signaling cascade. The c-fos is induced both by estrogens and anti-estrogens in ER-negative breast cancer cells [12, 15]. GPER-dependent proliferation of non-tumorigenic breast epithelial cells suggests GPER dependent estrogen-induced breast physiology and pathology [16]. The knockdown of GPER expression was shown to prevent the proliferation of triple negative breast cancer (TNBC) cells induced by E2 [17].

### Function of estrogen

Estrogen regulates early embryogenesis, in stage and tissue specific manner in zebrafish development [18]. Pluripotency-related genes, such as Oct4 and Nanog essential in embryogenesis are also regulated by estrogen. Knocking down of GPER decreases E2 induced proliferation both in embryo and in TNBC cells [17]. This explains that E2 might work via similar pathway both in embryo and in tumors despite of being TNBC. Embryogenesis and tumorigenesis may appear more or less as a same phenomenon to E2. Ovariectomy significantly decreased uterine weight which was recovered by 17β-estradiol administration. Ovariectomy impaired the growth of c-kit positive hematopoietic stem/progenitor cells (c-kit) into mixed cell type colonies. Whereas, in vitro condition the growth of colony remained unaffected in 17β-estradiol administered group. CD105-positive mesenchymal stem cells (CD105) in marrow were significantly decreased after ovariectomy [18]. The maintenance of stem cells specific for uterine growth is regulated by E2. Excess of E2 in post menopause may be significantly responsible for abnormal uterine growth through stem cells like c-kit and CD105.

Estrogen crucially maintains estrus cycle, female’s reproductive capacity, growth of egg follicles, fallopian tubes, endometrium and mammary gland. The question is how estrogen/estradiol involved in normal mammary gland development, turns into a carcinogenic substance. Estrogen, progesterone and prolactin, modulates the local expression of autocrine/paracrine growth factors [19, 20] such as Insulin like growth factor-1 (IGF-1), IGF-2, amphiregulin [19, 20] EGF, hepatocyte growth factor (HGF), transforming growth factor β (TGF-β), [21] heregulin, Wnt, RANKL and leukemia inhibitory factor (LIF) [22]. These factors responsible for mammary gland development regulates the activation of intracellular signaling cascades such as Erk, Akt, JNK, and Jak/Stat that control cell growth, proliferation and differentiation [23, 24]. The decision of a cell whether to renew, divide, differentiate or proliferate are consequences of external and internal signals (Table 1). E2 and its receptor is one of the important endogenous signals which regulates and determines the limits of a normal physiological condition. Breaking the upper limits of such endogenous and exogenous signals, may distort the balance among cell and influence cells decision of renewal, division, differentiation and proliferation resulting in tumorigenesis or cancer.

**Table 1:** Table summarizes estrogens associated regulatory molecules and their respective pathways delivering important physiological functions associated to breast cancer risk and initiating or promoting breast cancer

Mode of action of E2	Binding location	Regulated genes and pathways	Physiological function	Breast cancer risk
Genomic (direct) E2 + nuclear ER	Hormone response element on target gene	JUN, FOS, PGR, TP53, HRAS, Bcl2, BRCA1, CHAT, NQO1, CKB, LTF, SCGB1A1	Stress activated kinase, glucose homeostasis, cell growth and tissue development	These receptors, genes and pathways are associated to various cell growth associated functions The imbalances among these pathways may initiate or promote or support cancer
Genomic indirect E2 + nuclear ER	AP-1 (Jun/FOS) SP1 C-Rel subunit of Nfκβ ATF-2/C-Jun ATF-2/cAMP CREB JAK/STAT SRE ATF1/CREP Nuclear transcription factor Y	IGF-1, ovalbumin, cyclin D1 Retinoic acid-R 1α gene, LDL-R gene, eNOS, cfos, cyclin D1 Inhibits Nfκβ mediated expression of IL-6, inhibits cytokine IL-6 Cyclin-D1 Bcl2 gene E2F1 gene	Progression of cells through G1 phase of cell cycle Progression of cells through G1 phase of cell cycle Role in cytokine receptor signalling Anti-apoptotic	
Non-genomic E2 + ER	IGF-1 EGF G-protein SRC MMP	ERK P38/MAPK P13K/AKT PLC/PKC cAMP/PKA	Promotes G1-S promotes survival signal, enhances antiapoptotic Caspase3 eNOS activation-release NO	

### Molecular mechanism of estrogen, progesterone and testosterone associated breast cancer risks and therapy

Several anti-cancer drugs have been discovered which block important carcinogenic pathways. Anti-folate drug methotrexate (MTX) blocks dihydrofolate reductase (DHFR) that hampers folate pool. Blocking of folate pools hampers DNA synthesis and restricts tumor growth, especially in breast and ovary [25]. Interestingly proliferative action of estradiol (E2) in E2 dependent cancer does not interfere with folate function on cancer tissue in presence or absence of MTX [26]. MTX has been shown to alter several isoforms of sulfotransferase that potentially influence alcohol or steroids metabolism and drug–drug interactions [27]. Sulfotransferase regulated by MTX can be accountable during the treatment of E2 dependent gynecological cancers. Folate supplementation during MTX treatment has an influence on MTX associated induction of SULTS [28] and is implicated by selected nuclear receptors [29]. About progesterone and its role in breast cancer was associated with lower breast cancer risk compared to synthetic progestin when administered in combination with estrogen [30].

Hormone replacement therapy containing both estrogen and progestins increase breast cancer incidence while estrogen alone hormone therapy lowers breast cancer risk [31]. The full form (PRA/PRB) ratio is a prognostic and predictive factor for antiprogestin responsiveness in breast cancer [32]. Phosphorylation of Ser294 in a progesterone receptor is a common event in breast cancer progression, required to maintain stem cell fate in breast cancer [33]. Tumor levels of cytokeratin-5 (CK5) correlated positively with B Cell CLL/Lymphoma 6 (BCL6) in premenopausal women with hormone positive breast cancer. Elevated BCL6 or CK5 protein levels were associated with unfavorable clinical outcome. Progesterone (Pg) induces a CK5-positive basal cell-like population. Pg-induction of CK5 was preceded by upregulation of BCL6, an oncogene. Knockdown of BCL6 prevented Pg dependent induction of CK5-positive cell population [34]. Retinoid has been demonstrated to reduce the accumulation of CK5+ cells through retinoic acid receptor/progesterone receptor (RAR/PR) crosstalk during estrogen depletion. And this may explain the efficacy of retinoids in the prevention of breast cancer recurrences [35, 36]. Serum testosterone (T) may play important roles in the development of breast cancer in older women [37].

In contradiction some studies also report lower levels of bioavailable testosterone in women with breast cancers (BCA) [38]. Hence, Testosterone cannot be considered as the causative agent. Testosterone alone or in combination with anastrozole (A), delivered by subcutaneous implants, reduced tumor size and incidence of breast cancer, and was not associated with the recurrent breast cancer [39]. Testosterone is aromatized into estradiol and increased testosterone can be the causative agent in breast cancer via aromatization. Additionally both invasive and non-invasive breast cancer overexpresses aromatase leading to increased conversion of testosterone to estradiol [40, 41]. Testosterone along with anastrozole exerts a direct growth inhibitory effect by binding to the androgen receptor [42]. The three important hormones that is estrogen, progesterone, and testosterone were found to be associated with breast cancer (Table 2).

**Table 2:** Shows the list of SULT1E1 inducers

Inducers of SULT1E1	
Compound	Endogenous compound, receptor, transcription factors/pathways
Melatonin	Estrogen receptor and factors in hypothalamic–pituitary–reproductive axis
Diallyl sulphide (DAS)	Constitutive androstane receptor (CAR)
Dithiocarbamate derivative TM208	Estrogen receptor
Oxidative stress factors	Nuclear receptor factor 2 (Nrf2)
Progesterone	Progesterone receptors

### Genotoxic effects of estrogen metabolites and estrogen induced ROS

A number of studies have shown oxidative stress inducing capability of estrogen. 17β-hydroxysteroid dehydrogenase (17β-HSD) interconverts E2 and E1 [105]. During Phase I metabolism E2 and E1 are converted to catechol estrogens and 16α-hydroxyestrogens. The catechol estrogens (2-hydroxy or 4-hydroxyestrone/estradiol) produced through catalysis by CYP1A1 in the liver or CYP1B1 in tissues such as breasts, ovaries, and uterus [43].

The catechol estrogens can be converted into semiquinones and *o*-quinones by oxidizing enzyme or in the presence of transition metals such as Cu2+ or Fe3+ [44]. During these reaction procedures superoxide anion radicals and hydroxyl radicals are generated. These metal ions and hydroxyl radicals together participate in radical cascade reactions that finally damages DNA, lipids and proteins [45]. The catechol estrogen metabolites and E2 have almost comparable binding affinities to the ER. So catechol estrogen may induce estrogen-responsive gene expression via classical ER-mediated pathways [46, 47]. An *O*-methoxylated catechol estrogen induces proliferative effects and enhances tumor growth in animal models via genomic ER signalling pathways [48, 49]. Sulfation of E2 drastically impairs hormonal activities by facilitating their excretion. Sulfonated estrogens have minimum ER binding affinity whereas desulfation of estrogens facilitates active estrogen signaling in target tissues [50, 51]. Estrone sulfate is a major circulating metabolite and thought to be an important precursor of the active estradiol in postmenopausal women [52, 53]. Estradiol does not have any significant function in postmenopausal women and may undergo oxidative metabolism that produces reactive oxygen species (ROS) in cells [54].

Hydroxyl radicals are strong oxidizing agents that play a major role in oxidative damage to DNA bases. Treatment of E2 in hamsters induces various free radical-mediated oxidative damage including DNA single strand breaks [55, 56], formation of 8-Oxo-2′ deoxyguanosine (8-OHdG) [57], and chromosome abnormalities [58]. 8-OHdG being easily formed and highly mutagenic has been utilized as a biomarker of oxidative damage or carcinogenesis. Substantial evidence supports that the estrogen metabolites react with DNA, leading to the mutations responsible for the initiation of [57, 58]. A reducing environment in the presence of a low level of ROS is beneficial to normal cellular process including signal transduction, apoptosis, cell differentiation, and regulation of transcription factors [59, 60]. However, excess ROS could chemically modify cellular macromolecules including DNA, proteins, carbohydrates, or lipids, thereby disrupting normal physiological functions of these biomolecules [54]. Estrogen-mediated oxidative DNA damage in the epithelia of mammary tissues results in the induction of 8-oxo-2′-deoxyguanosine. It may indicate the pro-oxidant effects of estrogen [61]. E2 is also capable of inducing an increase in oxidative DNA-damage through an ER-mediated mechanism [62]. One-electron oxidation of estradiol may generate reactive phenoxyl radical intermediate. These metabolites abstract hydrogen from reduced glutathione generating the glutathione thiyl radical [63]. Similarly, the estradiol phenoxyl radical abstracts hydrogen from reduced β-nicotinamide-adenine dinucleotide (NADH) resulting in the generation of the NAD· radical. These reaction steps finally produce superoxide and its dismutation product, hydrogen peroxide and further propagate a chain reaction of group of free radical cascade [64].

The accumulation of intracellular hydrogen peroxide could explain the hydroxyl radical-induced DNA base lesions in female breast cancer tissue [65]. This “estrogen-induced oxidative stress” may directly affect the redox-sensitive transcription factors such as nuclear factor-erythroid-2-related factor 2 (Nrf2), activating protein 1 (AP-1), or NF-κB transcription factor. All these are involved in mediating inflammatory responses and key players in carcinogenesis [66].

### Redox regulated enzymes/receptors are involved in direct and indirect regulation of estrogen

#### Estrogen sulfotransferase regulations under oxidative stress

SULT1E1 catalyzes the sulfoconjugation of estrogens at the 3′-hydroxyl position at nM concentration as compared to other sulfating enzymes such as phenol sulfotransferase (SULT1A1) which sulfoconjugates at µM concentration of E2. SULT1E1 has 300-fold higher affinity for estrogen sulfation than SULT1A1 [67]. Ethanol may alter SULT1A1 and SULT2A1 (steroids mainly DHEA catabolizing enzyme) proteins and their enzymatic activities. Though differing in their substrate specificity, but due to overlapping substrate preferences in SULT families may modulate their substrate utilization both qualitatively and quantitatively [68]. The binding affinity of E2 to ER is twice as high as that of E1. While sulfoconjugated estrogens i.e., (estradiol 3-sulfate) E2S and (estrone 3-sulfate) E1S show no ER binding activity [69]. And so sulfoconjugation may be one of the direct methods of deactivating E2. Since E2 is a strong breast cancer causative agent, E2 metabolizing proteins or associated receptors and proteins also become important to be kept under surveillance.

SULT1E1 activity is found to be significantly declined during the process of breast carcinogenesis, MCF7 cells transfected with EST expression vector incubated with 20 nM E2 showed significantly rapid sulfation of E2 than the control, which was transfected only with vectors [70]. SULT1E1 is responsible for sulfating active 17β-estradiol (E2) into an inactive form. In recent years, the correlation between SULT1E1 and estrogen-dependent cancers has been noticed. Report reveals that SULT1E1 level is inversely correlated with the degree of malignancy in breast cancers [71]. It is suggested that E2S level is lower than E2 in breast cancer, indicating low SULT1E1 activity. These studies suggest the carcinogenesis process reduces SULT1E1 and so induction of SULT1E1 may reverse the situation [72]. We have discussed that estrogen induces oxidative stress via ROS generation [60]. An earlier in vitro study from our lab suggested a potential oxidative regulation for hSULT1E1 through the redox modification of Cys-83 [73]. The number cysteine-83 is located in the active E2 binding site. The thiol (–SH) group of Cys83 is directed towards the E2 molecule based on its crystal structure. Cys83 modification by –SG was sufficient to inactivate hSULT1E1, probably by inhibiting substrate binding or product release [73]. Some reports which says oxidative stress increases either E2 or decreases SULT1E1. To the best of our knowledge, oxidative regulation of human SULT1E1 in human breast cancer has not been reported. Redox regulation of SULT1E1 as reported in in vitro studies may also attributes to low SULT1E1 activity in breast cancer.

#### Inducers of estrogen sulfotransferase

Induction of SULT1E1 in breast cancer patients may provide new treatment strategies. So, investigating studies of the inducer of SULT1E1 is also a necessity. Oxidative stress induces SULT1E1 through Nrf2 activation, SULT1E1 is a direct transcriptional target of Nrf2 [74]. Interestingly, estrogen also regulates the expression and activity of Nrf2. Thus, a cyclic regulatory mechanism is generated where the Nrf2 induces the expression of SULT1E1, which increases deactivated estrogen and restricts the estrogen-responsive activation of Nrf2. NRF2 pathway creates an environment that favors the survival of normal as well as malignant cells, protecting them against oxidative stress. The expression of SULT1E1 in the tumor microenvironment may also become unfavourable for the cancer cells as it will restrict the estrogen dependent activation of Nrf2. Progesterone possesses an agonistic effect on SULT1E1 transcription. SULT1E1 was concentration dependently antagonised by progesterone receptor modulators (SPRMs) mifepristone (RU486) and apigenin [75]. The induction of SULT1E1 was inhibited by RU486 indicating a role for the progesterone receptor. Melatonin serves as an endogenous antioxidant. Melatonin suppresses cell proliferation during breast cancer by inhibiting the up regulation of estrogen-induced cyclin D1. Here cyc-D1 is induced via G-protein-coupled receptor. Melatonin stimulates the expression of SULT1E1 [76]. Melatonin may target cell cycle arrest by upregulating SULT1E1. Diallyl sulfide (DAS), a component of garlic induces estrogen sulfotransferase. The SULT1E1 gene in mouse liver is expressed through regulation of xenobiotic receptor constitutive androstane receptor (CAR). There was no decrease in serum levels of endogenous E2 or increase in estrone sulphate, but the clearance of exogenously administrated E2 was accelerated in DAS treated mice [77]. So, CAR mediated SULT1E1 induction may be utilized to control E2 induced breast cancer. Oxidative stress and free radicals directly (by protein modification) or indirectly (suppressing SULT1E1 transcription) inactivate the enzyme activity. Both endogenous and exogenous antioxidants may inhibit the enzymatic suppression by reducing oxidant stress.

#### Stress regulations of estrogen receptor-α

Seventy percent of the total breast cancers overexpressing ER alpha responds to anti-estrogen (for example tamoxifen) therapy. Tamoxifen regulates several steroids metabolizing gene in female rats and suggests, a re-evaluations of the exact efficacy of tam in disease therapy with both qualitative and quantitative approaches [78]. ERα is downregulated when exposed to oxidative stress induced by H_2_O_2_ in MCF 7 cells. This down regulation was not due to proteosomal degradation pathway, but due to decrement in mRNA level resulting in an impaired estrogen signaling. Redox-dependent modifications of ERα in its Cys rich DNA binding domain are also noticed. The sulfhydryl groups within the two Cys4 zinc fingers of the DNA-binding domain (ER-DBD) of estrogen receptors are modified resulting in structural damage, hence a loss in ER’s DNA-binding capacity. Loss of ER’s DNA binding capacity is observed in many ER-positive breast cancers. It is very likely contributes to an altered transcriptional activity during human breast cancer pathogenesis [79]. A study reports that the cellular content of full form (TRX) determines the transcriptional activity of ER. This indicates that the function of ER is highly sensitive to the cellular redox state [80]. In a breast cancer model, antioxidant cellular capacity is modulated through ER activity. This data may explain some of the estrogen induced pro-oxidant effects as previously reported in an in vivo study. Report reveals that E2 is capable of inducing an increase in sensitivity to oxidative DNA damage through an ER-mediated mechanism [50]. Thus, oxidative stress alters the structure and function of human ERα isoform through cysteine modification which leads to effective transcriptional alteration via E2 signaling.

#### Stress regulations of sulfatase

Steroid sulfatases (STS) convert estrone sulfate (E1S) and dehydroepiandrosterone sulfate (DHEAS) to estrone (E1) and dehydroepiandrosterone (DHEA), respectively. Estrone and DHEA may then be used for the synthesis of estradiol which eventually fuels the ERα+ breast cancer cells [51]. The activity of steroid STS is much higher than aromatase in breast tumors. Increased STS mRNA in tumors is associated with poor prognosis of this disease. Inhibition of STS activity was associated with significant reductions in serum concentrations of estrogens and androstenedione, resulting in disease stabilization. Targeting STS can be a potential treatment for hormone-dependent breast cancer in postmenopausal women [81]. In hormone dependent tissues, local E2 is more effectively synthesized from estrone sulfate, than via the aromatase pathway [82]. STS converts E1S to estrone, which is then converted to E2 by 17β-HSD [83]. The expression of tissue-specific STS is controlled by ERα signaling in both normal and cancerous breast tissue [84]. More active expression of STS isoform may occur under estrogen therapy in patients with ERα positive breast cancer. STS will again upregulate estrogen, which would further promote cancer progression [82]. E1 is converted to E2 by 17β-hydroxysteroid dehydrogenases (17-βHSDs). Thus, the increased expression/activity of STS paralleled with increased 17-βHSDs may lead to an increased production of active E2.

Sulfatases requires a modification of the cysteine residue present at the catalytic site of the enzyme, to become active. In eukaryotes this cysteine is converted into formylglycine (FGly) by formylglycine generating enzyme (FGE) by oxidation. The prokaryotic sulfatase carries a serine residue at their catalytic site which undergoes modification. This oxidation occurs shortly after the import of the nascent sulfatase polypeptide in the endoplasmic reticulum. CTPSR is the short linear sequence in the sulfatase that directs the binding of the FGE [85]. Multiple sulfatase deficiency (MSD), a fatal autosomal recessive syndrome has been regarded a critical pathological conditions noticed in several human [86]. High-resolution structures of FGly-generating enzyme (FGE) in different redox environments was resolved and reported. A novel oxygenase mechanism has been demonstrated where FGE utilizes molecular oxygen to generate FGly via a cysteine sulfenic acid intermediate. FGly the key catalytic residue in the active site is unique to sulfatases [87]. Aromatase inhibition in postmenopausal women is a well-established treatment of hormone dependent breast cancer. But the endocrine resistance may cause progression of the disease towards metastasis. Several alternative enzymes involved in steroid synthesis and metabolism have recently been investigated as possible drug targets. Steroid sulfatase can be one of the most efficient targets in the treatment of breast cancer.

#### Stress regulations of formylglycine generating enzyme

Formyl glycine generating enzyme (FGE) executes the vital activation step of all sulfatases and sulfatases then executes a critical activation step of estrogen desulfation. FGE is localized in the endoplasmic reticulum and modifies the unfolded form of newly synthesized sulfatases [87]. The generation of FGly from a cysteine residue is a multistep redox regulated process that involves disulfide bond formation [88]. This process also requires the molecular oxygen, but does not require any cofactors or metal ions. Other peptides including hSTS that contain the minimal motif CTPSR (cysteine 69, Threonine 70, proline 71, serine 72 and arginine 73) with flanking sequences are substrates for FGE and are converted to their FGly-containing counterparts [89]. The amino acid position 70 in STS shows variability, being occupied either by threonine, serine, cysteine or alanine. The minimal motif CTPSR is conserved in all human sulfatases suggesting a general binding mechanism of substrate sulfatases by FGE [85, 86]. Since, FGE oxidises a cys thiol in sulfatase into an aldehyde and this conversion might be favoured by an oxidising environment. Thus, FGE has an important role in the regulation of estrogen through oxidation of sulfatase. If FGE itself is inactive, then sulfatase will be inactive and will not process E1S/E2S into estrone and estradiol. Conclusively, it can be inferred that SULT1E1 is active when its cysteine is in a reduced state opposite to sulfatase which gets activated when one of its cysteine is oxidised by FGE. Mutations in sulfatase gene (SUMF1) result in defective FGE leading to impairment of sulfatases. This event occurs in multiple sulfatase deficiency (MSD), in which early infant death occurs due to the accumulation of glycosaminoglycan’s or sulfolipids can cause [90, 91].

## Discussions

**Fig. 1:**
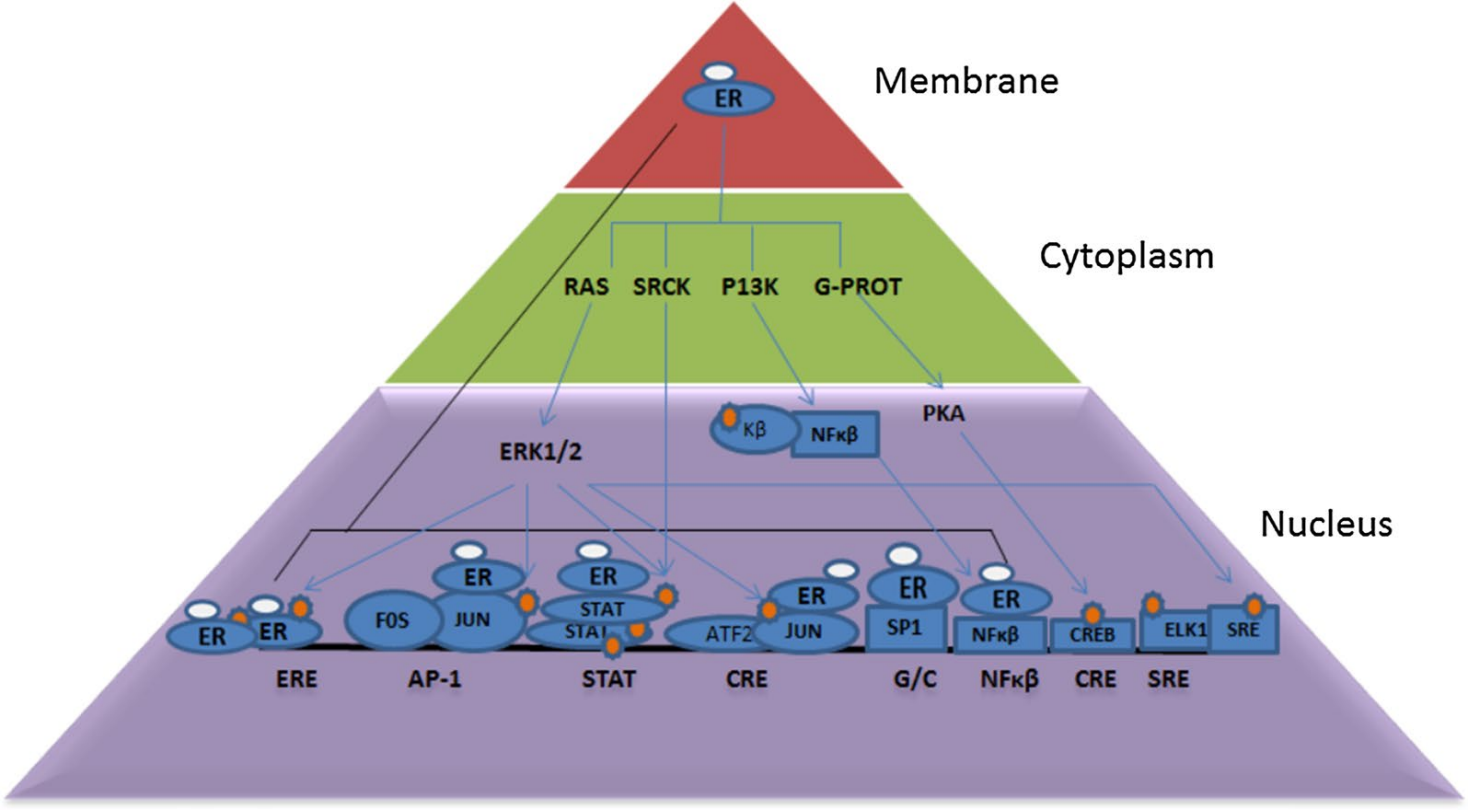
E2-ER complexes binds to EREs and also to transcription factor complexes, e.g. AP-1, STATs, ATF2 (activation transcription factor 2)/c-Jun, Sp1, and NFκβ that are already bound to their specific DNA binding sites Membrane E2-ER complexes activate protein-kinase cascades, leading to phosphorylation (P) of target transcription factors, e.g. AP-1, STATs, Elk-1, SRF (serum response factor), CREB and NFκB. The phosphorylation results in their transcriptional activation or modulation of the transcriptional activities of ER-AP-1, ER-STAT, ER-Sp1, and ER-NFκB complexes. Protein-kinase phosphorylates ERs resulting in ligand independent transcriptional activity

**Fig. 2:**
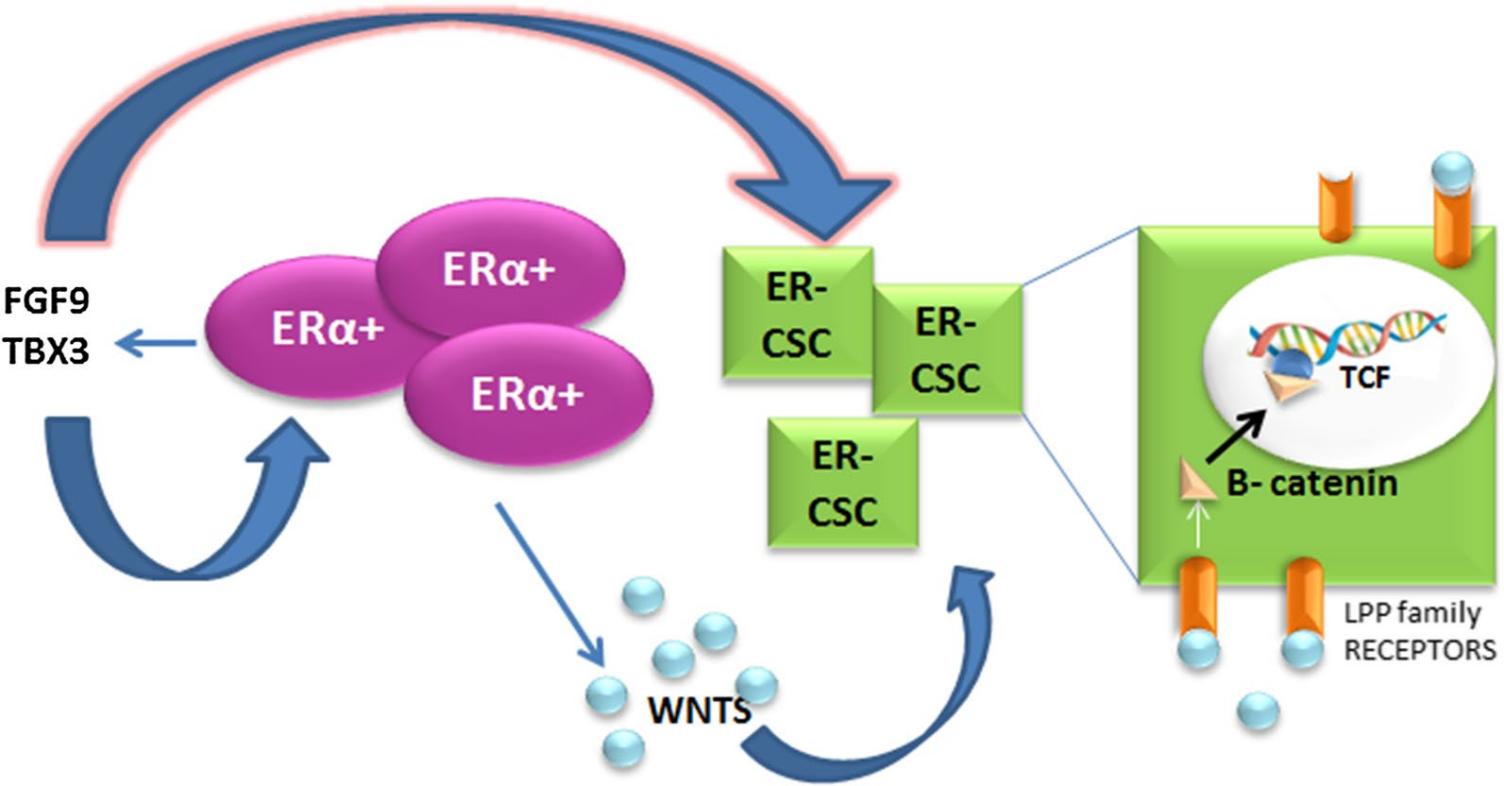
ERα+ cells expresses FGF9 TBX3, TBX3 further expresses FGF and Wnt, Wnts bind to LRP receptors which transduces a signal to β-catenin, β-catenin binds to TCF to transcript Wnt genes

**Fig. 3:**
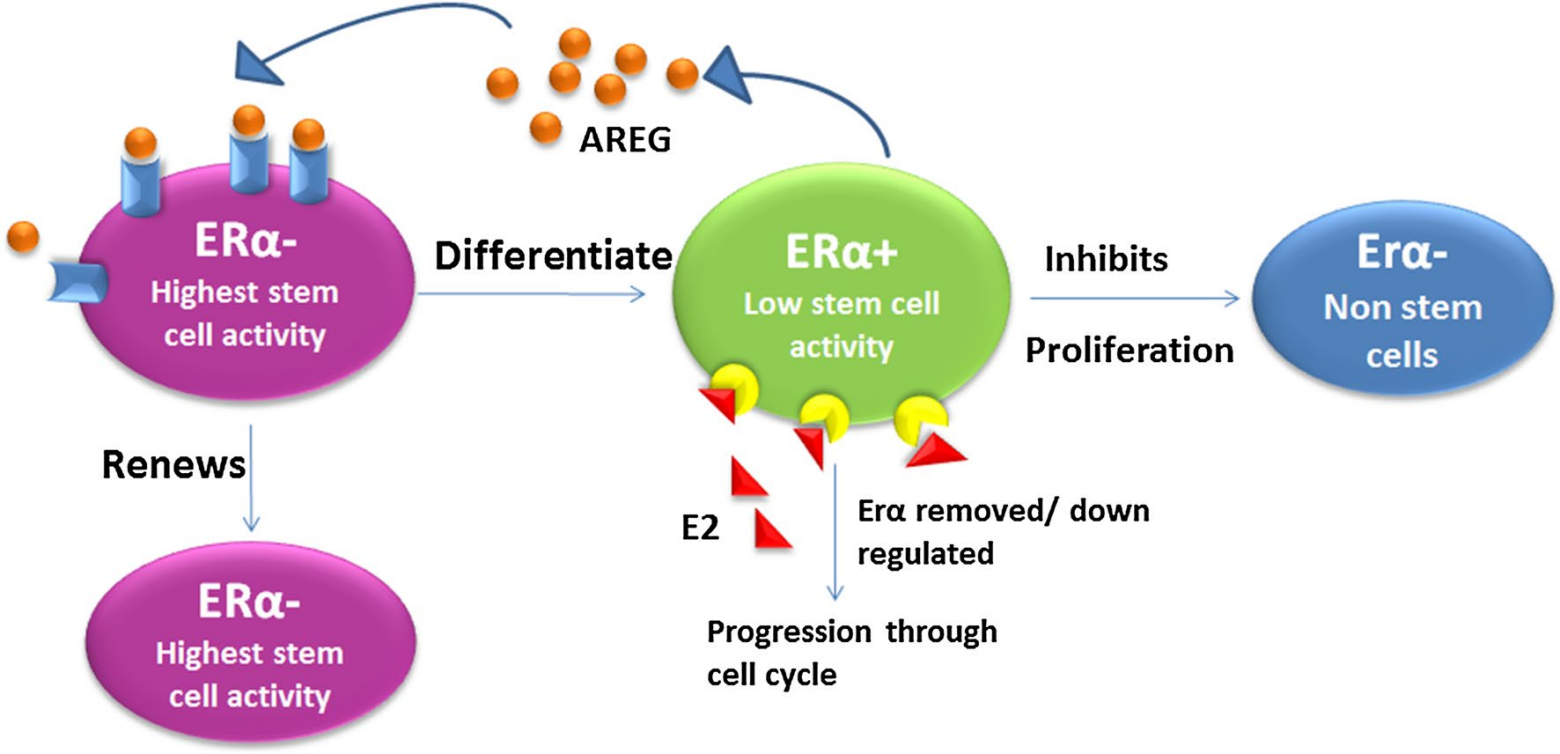
E2 controls the ER− stem or non stem cell in a paracrine manner, the self renewal of Erα− stem cells and its differentiation into Erα+ cells depends on amphiregulin secreted by Erα+ cells. Presence of ER+ stem cell inhibits proliferation of Erα− stem cells

Nature has selected estrogens for various functions in both male and female starting from the very beginning of the embryogenesis. Later, estrogens become specifically more important in females conducting functions like embryonic and pubertal mammary gland development, maintenance of the estrus cycle, and organs like ovary and uterus (including morphogenesis and organogenesis). Estrogen delivers its function via estrogen receptors both in genomic and non-genomic pathways, which convergely expresses estrogen receptor element (ERE) or activates various transcription factors which are bound to their cognate DNA sequences (Fig. 3). Eventually, pathways like Srck, P13K, MAPK and transcription factors like AP-1, STAT, SRE and SP1 are activated (Fig. 1 and Table 1) resulting in expression of paracrine and autocrine growth factors like EGF, FGF, HGF, amphiregulin, Wnt (Fig. 2) etc. These factors regulate cell proliferation and differentiation. Although E2 is a vital molecule in females, estrogen was found to be tumorigenic in both pre and post menopausal women, depending on factors like early menarche late pregnancy, late menopausal and obese postmenopausal BMI. All these factors infer that long term exposure to estrogen and other steroid hormone leads to tumor.

Obese post-menopausal women were found to be associated with increased breast cancer risk because of high concentration of prevailing estrogen as cellular proliferators. In some post-menopausal cases of abnormal steroid metabolism, estrogen levels remain high in the blood. But at this stage reproduction and associated functions become ceased. An elevated level of E2 initiates unnecessary growth in the reproductive tissues and eventually leads to tumorigenesis. Estrogen regulates breast cancer stem cells through fibroblast growth factor, its receptor and T-box transcription factor (FGF/FGFR/TBX3) (Fig. 2). Estrogen signaling via FGF/FGFR/TBX3 is also noticed in normal mammary gland development, during embryonic and pubertal stages in females. Estrogen induces synthesis of amphiregulin (a ligand of epidermal growth factor receptor (EGFR)) in ERα+ non-stem cell. Amphiregulin then stimulates the proliferation of ERα− stem cell which in turn differentiates into both ERα+ and ERα− stem cells (Fig. 3). Thus, the cellular proliferation and differentiation of ER− and ER+ is regulated by ER+ cells.

**Fig. 4:**
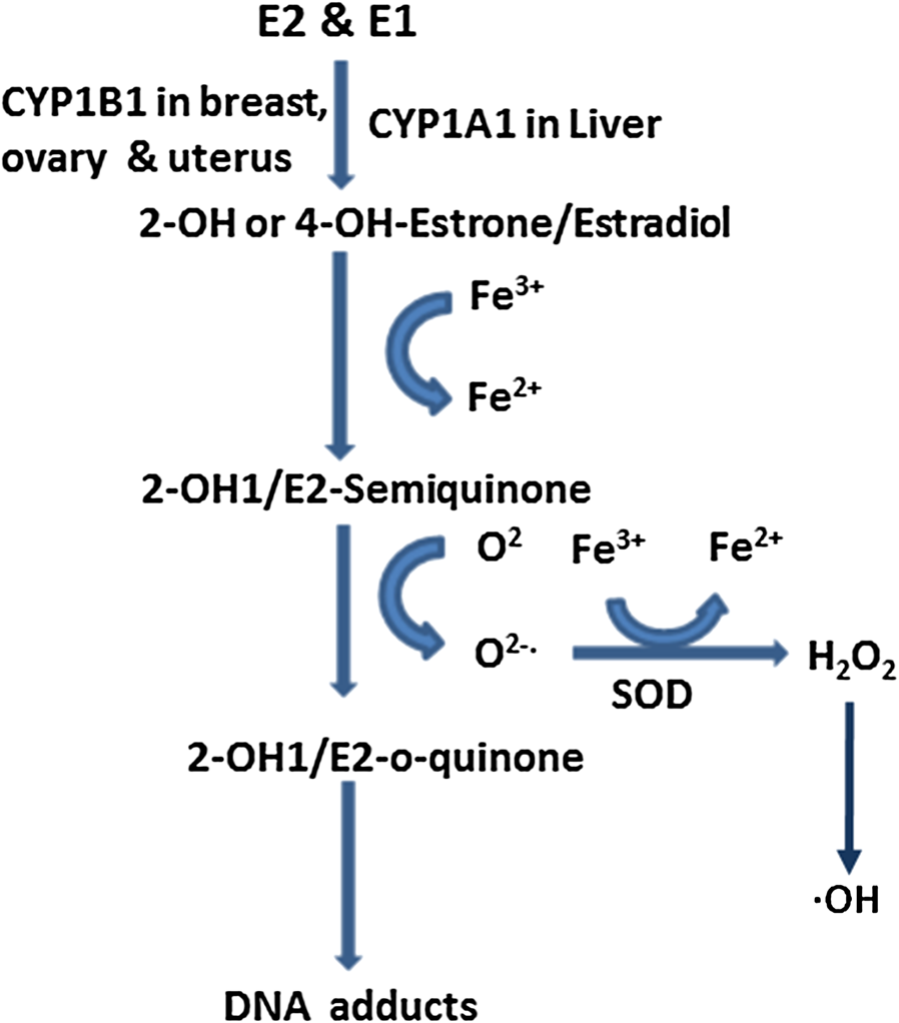
Estrogen induces production of ROS when semiquinones are converted to *o*-quinones

Estrogens activity is not limited to ER+ cells, but by induction of some intermediate molecules like amphiregulin estrogen can control beyond ER+ cells. Amphiregulin is an important molecule for ductal morphogenesis during pubertal mammary gland development. Thus estrogen mediated amphiregulin induction plays an important role. Reports reveal that estrogen in epithelial tumors exerts malignant transformation of immature ovarian teratoma through a non-genomic pathway. Cancer antigen 125 (CA125) a dependable marker of gynecological cancer is also associated to estrogens increased levels. Hormone replacement therapy (HRT) with estradiol showed persistently increased levels of CA125 which returned to the normal level after discontinuation of the therapy. This suggests that estrogen may regulate CA125 [92]. Both malignant epithelial ovarian tumors and advanced breast cancers have shown significantly higher CA125 along with high estradiol, enhanced cellularity/histo-architectural impairment/unstable-DNA [93]. Other than cellular proliferation estrogen is found to induce oxidative stress through ROS generation (Fig. 4). Stress is the most obvious by-product of modern day life. Stress can be generated in the system exogenously or endogenously. Physiological and systemic stress generated by endobiotics and/or xenobiotic can initiate several important metabolic syndromes. Abnormal regulations of steroids, monoamines or other biomolecules like cortisol, adrenaline may generate serious disease like cancer and diabetes. In addition, environmental pollutants are one of the unavoidable ingredients of the present day. Pollutants include heavy metal contaminants such as arsenic and different types of pesticides, herbicides, environmental exhausts and industrial wastes. More than 80% of the world populations are exposed to one or more of these stress generating pollutants. Reports reveal that a large number of populations are experiencing and in the near future may be affected by different types of disease like cancers and type-2 diabetes.

**Fig. 5:**
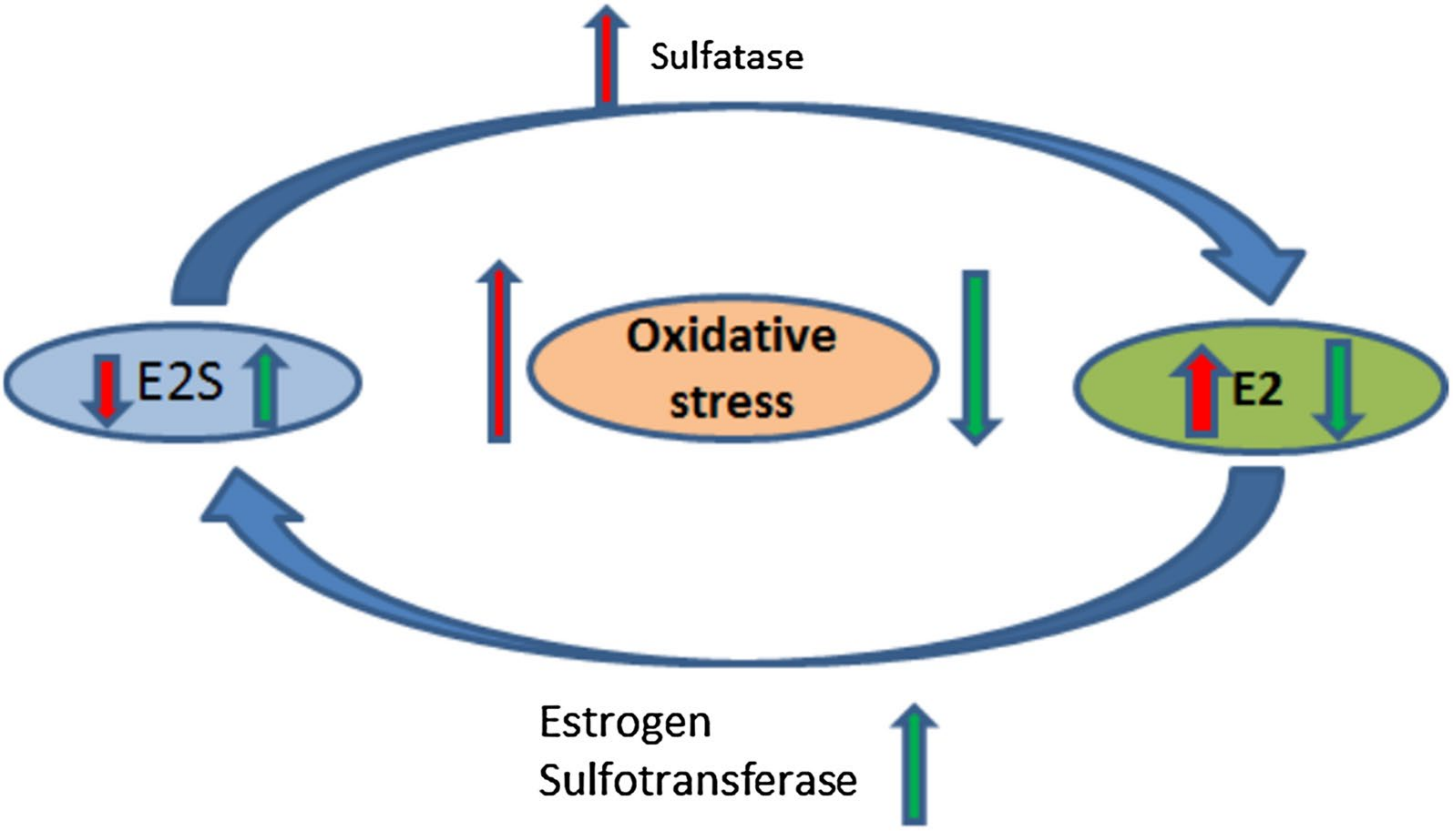
High oxidative stress upregulates Sulfatase and downregulates estrogen sulfotransferase resulting in high E2 and low E2s and vice versa

Oxidative stress associated regulations of different SULTs isoforms have been decisively demonstrated earlier [94, 95]. This review conclusively found stress (estrogenic or non-estrogenic) favors activation and inactivation of various estrogen regulating molecules such as estrogen sulfotransferase, estrogen receptor, sulfatase and FGE (Fig. 5). A reducing environment favors to keep SULT1E1 and ER functionally active. Inversely, sulfatase and formylglycine generating enzyme requires an oxidizing environment to become active. Under oxidative stress ERα was down-regulated at mRNA level and ERα also undergoes redox-dependent modifications at the Cys rich DNA, and loses its DNA binding capacity. Whereas, Sulfatase, which converts E1S and E2S into active and functional E1 and E2 need to be oxidised at its catalytically active site. The thiol of a cysteine residue is oxidized into an aldehyde leading activation of STS. It is not very clear from the earlier studies whether this activation of the sulfatase is favored by oxidative stress or not. Though the FGE is responsible for sulfatase activation requires molecular oxygen to conduct its function. Further studies are required for the clarification. The pathways activated by oxidative stress such as thymidine phosphorylase, hypoxia activating kinase which stimulates the expression of chemoattractants (endothelin 2, or rapid neoplastic growth and metabolism (BOX-2)) are the pathways associated to immortalization and malignant transformation. These pathways are correlated with clinical prognosis in breast cancer patients. The question is how oxidative stress alter the aspects of tumor biology, such as the endocrine pathways that drive the occurrence of ERα+ breast cancer [96]. Some mechanisms relating oxidative stress and pathogenesis of breast cancer have been elucidated. No systematic review has yet been done on redox regulation of estrogen metabolizing proteins and interference in estrogens signaling. In this regard the present review focuses on some novel aspects of this issue.

## Conclusions

Thus, the ability of estrogens to generate oxidative stress is more or less like “self -help”. Where sulfatase is highly active to produce active estrogen but ERα gets down regulated both at mRNA level and functional level. Estrogen receptor becomes unable to bind to DNA due to structural modification resulting in a balanced E2 signaling. Possibilities have been explored that SULT1E1 activity severely impairs during the process of carcinogenesis. Later, an in vitro studies reported that SULT1E1 is also redox regulated where a cysteine (position − 83) present at E2 binding site can be modified, inhibiting E2 sulfoconjugation. Though SULT1E1 being inactive this “self-help” via ER may just marginally sufficient to maintain a normal physiological condition. The tendency to be abnormal still remains high since SULT1E1 remains inactive under oxidative stress. Accordingly, if once estrogen has induced cancer; functional inability of ER would not be able to compensate the availability of E2 favored by low SULT1E1 and high sulfatase under oxidative stress. Hence, SULT1E1 plays a vital role in lowering the level of active estrogen, both in normal and cancerous state. Further studies are required to investigate whether the estrogen availability is significantly affected by SULT1E1 and to see if the same redox regulation of SULT1E1as in vitro is responsible for impairment of SULT1E1 activity in vivo. Although reports reveal that reactive oxygen contributes to the age-related cancers, especially ER dependent breast cancer. No definitive carcinogenic mechanisms have been reported [97]. The vicious circle between the generation of carcinogenesis by oxidative-stress and vice versa complicates the understanding of the role of oxidative stress in the generation of Erα+ breast cancer. Present review highlights new aspect on the role of oxidative stress in the redox-modification of E2-regulating proteins which finally modulate E2 signaling in cell division/transformation and carcinogenesis. We strongly hope this review will be helpful for researchers and clinicians for a better realization of disease mechanism and its therapeutic approach.

## Abbreviations

ER: estrogen-receptor
SULT1E1: estrogen-sulfotransferase
STS: sulfatase
FGE: formylglycine-generating-enzyme
ERT: estrogen replacement therapy
IAR: International Agency for Research
NIEHS: National Institute of Environmental Health Sciences
AP-1: activator protein1
STATs: signal transducer and activator of transcription
ATF-2: activator of transcription factor 2
NFκβ: nuclear factor kappa-light-chain-enhancer of activated B cells
TNBC: triple negative breast cancer
IGF-1, IGF-2, EGF: insulin like growth factor-1
HGF: hepatocyte growth factor
TGF-β: transforming growth factor β
LIF: leukemia inhibitory factor
MTX: methotrexate
DHFR: dihydrofolate reductase
E2: estradiol
RAR/PR: retinoic acid receptor/progesterone receptor
DAS: diallyl sulfide
CAR: constitutive androstane receptor
DHEAS: dehydroepiandrosterone sulfate
17-βHSDs: 17β-hydroxysteroid dehydrogenases
CA125: cancer antigen 125
